# Oligoribonucleotide interference-PCR: principles and applications

**DOI:** 10.1093/biomethods/bpac010

**Published:** 2022-05-13

**Authors:** Takeshi Shimizu, Toshitsugu Fujita, Hodaka Fujii

**Affiliations:** Department of Biochemistry and Genome Biology, Hirosaki University Graduate School of Medicine, Hirosaki, Aomori 036-8562, Japan

**Keywords:** ORNi-PCR, PCR, mutation, CpG methylation, polymorphism

## Abstract

Polymerase chain reaction (PCR) amplification of multiple templates using common primers is used widely for molecular biological research and clinical diagnosis. However, amplifying a specific DNA sequence harboring a mutation that is present in a small number of mutant cells within a large population of normal cells (e.g., as in cancer) in a tissue is difficult using the original PCR protocol. Thus, some measures are necessary to suppress amplification of background signals. To achieve this, we developed the oligoribonucleotide (ORN) interference-PCR (ORNi-PCR) technology in which an ORN (short RNA) hybridizes with a complementary DNA sequence to inhibit PCR amplification across the specific target sequence. ORNs can be prepared inexpensively, and ORNi-PCR can be carried out easily by adding ORNs to the PCR reaction mixture. Suppressing amplification of target sequences by ORNi-PCR is useful for detecting target sequence mutations. We showed that ORNi-PCR can discriminate single-nucleotide mutations in cancer cells and indel mutations introduced by genome editing. We also showed that ORNi-PCR can identify the CpG methylation status of a target sequence within bisulfite-treated DNA, and can enrich DNA sequences of interest from a DNA mixture by suppressing amplification of unwanted sequences. Thus, ORNi-PCR has many potential applications in various fields, including medical diagnosis and molecular biology. In this review, we outline the principles of the ORNi-PCR method and its use to detect nucleotide mutations in a variety of specimens.

## Introduction

Sanger sequencing is a reliable method for detecting mutations in genomic DNA (gDNA); however, it takes a long time and has low sensitivity. Next-generation sequencing (NGS) is a nonbiased method to identify mutations; however, it is too expensive to simply identify defined mutations.

Polymerase chain reaction (PCR) is a standard method for amplifying nucleotides for analysis and use in various fields such as clinical diagnosis [1]. Although PCR can amplify target sequences specifically, annealing of primers to nontarget sites will amplify nontarget templates. In addition, when a wild-type (WT) DNA with a high copy number and mutant DNA with a low copy number are both amplified by PCR using a certain primer set, selective amplification of only the mutant DNA is difficult. Various improved PCR methods have been developed to specifically amplify only the target DNA sequences [2]. Blocking PCR enables detection of specific DNAs and identification of nucleotide mutations by suppressing amplification of nonspecific amplicons [2]. Blocking PCR utilizes 3′-modified DNAs and artificial nucleic acids such as peptide nucleic acids (PNAs) and locked nucleic acids (LNAs) complementary to nondesired target DNA, and these tools block extension by DNA polymerase or compete with annealing primers [3, 4]. While such artificial nucleic acids may have the advantage of exhibiting affinity for target DNA sequences, resistance to nucleases, or higher stability, they also have a disadvantage in that they are more expensive to synthesize than 3′-modified DNAs.

## Development of the ORNi-PCR method

**Figure 1:**
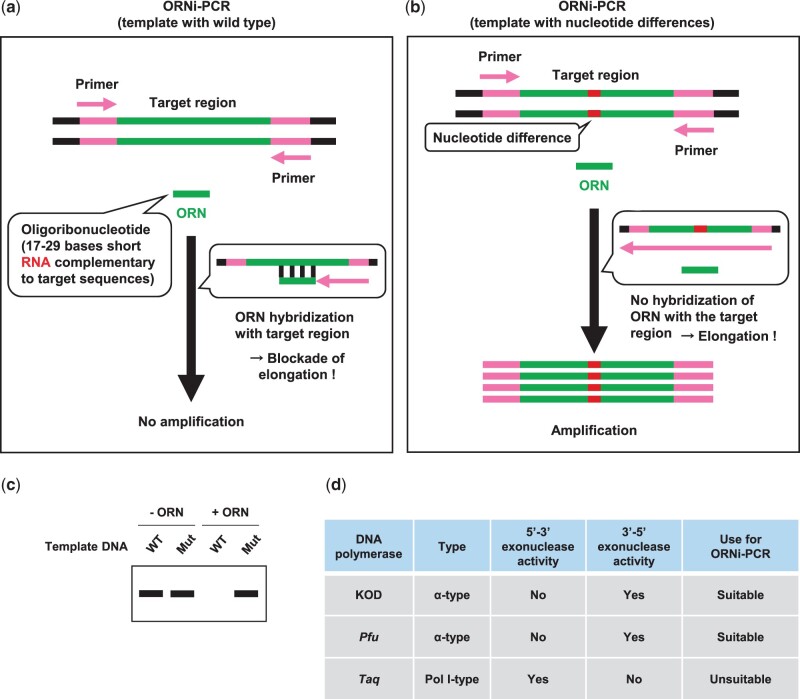
The principle of ORNi-PCR. (a) In ORNi-PCR, an ORN (a short RNA complementary to target sequences) hybridizes with a target site and blocks elongation of strand synthesis by a DNA polymerase that does not have 5′–3′ exonuclease activity. (b) If a target site is mutated, an ORN cannot hybridize to that target site, resulting in amplification of the target. (c) Image of a gel showing the results of ORNi-PCR. In the absence of an ORN (−ORN), the target region is amplified from both WT and mutated (Mut) DNA templates. However, in the presence of an ORN (+ORN), amplification of the WT is suppressed. (d) The properties of KOD, *Pfu*, and *Taq* DNA polymerases, and their suitability for ORNi-PCR.

Previously, we developed oligoribonucleotide (ORN) interference-PCR (ORNi-PCR), an ORN-based blocking PCR method that uses ORNs to block PCR amplification across specific target sequences [5]. In ORNi-PCR, an ORN (usually 17–29 bases long) containing sequences complementary to that of the target region hybridizes to the target region to block and inhibit its amplification (Fig. 1a). If a target DNA sequence contains nucleotide differences (e.g., a single-nucleotide mutation) then the ORN cannot hybridize to the target DNA sequence and the target region is amplified (Fig. 1b). After ORNi-PCR, electrophoresis of the ORNi-PCR amplicons can determine whether differences exist in the target DNA sequence (Fig. 1c). Previously, we compared ORNi-PCR using KOD, *Pfu*, and *Taq* DNA polymerases [5]. In the presence of KOD and *Pfu* polymerases, but not *Taq* DNA polymerase, amplification of target DNA was suppressed by an ORN. KOD and *Pfu* polymerases lack 5′–3′ exonuclease activity (i.e., α-type), whereas *Taq* DNA polymerase retains this activity (i.e., Pol I-type). These results suggest that 5′–3′ exonuclease activity may remove an ORN that is hybridized to its target DNA sequence. Therefore, α-type DNA polymerases such as KOD and *Pfu* polymerases can be used for ORNi-PCR (Fig. 1d) [5]. Elongation by such DNA polymerases can be blocked by ORNs, although ORNs themselves do not work as primers; however, there are some DNA polymerases that can amplify DNA using RNA primers *in vitro* [6]. That said, amplification of DNA by RNA primers is not efficient and, usually negligible compared with the exponential amplification afforded by DNA primers [6]. ORNs can be synthesized cheaply, which is a big advantage over PNAs and LNAs. In addition, the design of an ORN is flexible [5], whereas that of a PNA may be restricted in some cases [7], which is also an advantage of ORNi-PCR. DNA modified at the 3′-position to block extension can also be used as a cost-effective nucleotide blocker. However, DNA polymerases possessing 5′–3′ exonuclease activity degrade hybridized 3′-modified DNA during DNA extension. DNA polymerases possessing 3′–5′ exonuclease activity (proofreading activity) will remove the 3′-modification, leading to undesirable DNA extension from its 3′-position. Therefore, the selection of DNA polymerases is a considerable issue when using 3′-modified DNA as a blocker. In general, RNA–DNA hybrids are more stable than DNA–DNA hybrids, which is also an advantage of an ORN over 3′-modified DNA. Since (i) synthesis of an ORN is less expensive, (ii) one can design an ORN more flexibly, and (iii) an optimization protocol for ORNi-PCR has been established (see below), we believe that ORNi-PCR is an easier method to use.

In contrast, the melting temperature of LNA–DNA and LNA–RNA strands is higher than that of RNA–DNA and DNA–DNA strands [8]. In addition, ORNi-PCR uses DNA polymerases lacking 5′–3′ exonuclease activity because this activity may degrade an ORN hybridized to a target sequence during DNA extension. In this context, *Taq* DNA polymerases retaining the 5′–3′ exonuclease activity cannot be used; therefore, real-time target detection in combination with a TaqMan probe would be challenging for ORNi-PCR at this stage, which may be considered a limitation. However, ORNi-PCR would be still advantageous if gel electrophoresis or real-time target detection, in combination with SYBR Green I, is used to confirm DNA amplification.

In general, PCR comprises three steps: denaturation, annealing, and elongation. Initially, we showed that nucleotide differences can be detected by the three-step ORNi-PCR method [5, 9]. First, to detect genome-edited cells by ORNi-PCR, it is necessary to design an ORN that hybridizes with the target site used for genome editing. Next, the ORN is added to the PCR reaction solution and ORNi-PCR is performed using gDNA prepared from the genome-edited cells as a template. If a mutation is introduced correctly into the target site by genome editing, the target DNA will be amplified because the ORN cannot hybridize. In other words, by examining whether or not the target DNA is amplified by ORNi-PCR, it is possible to determine whether or not the target site has been edited [9].

**Figure 2:**
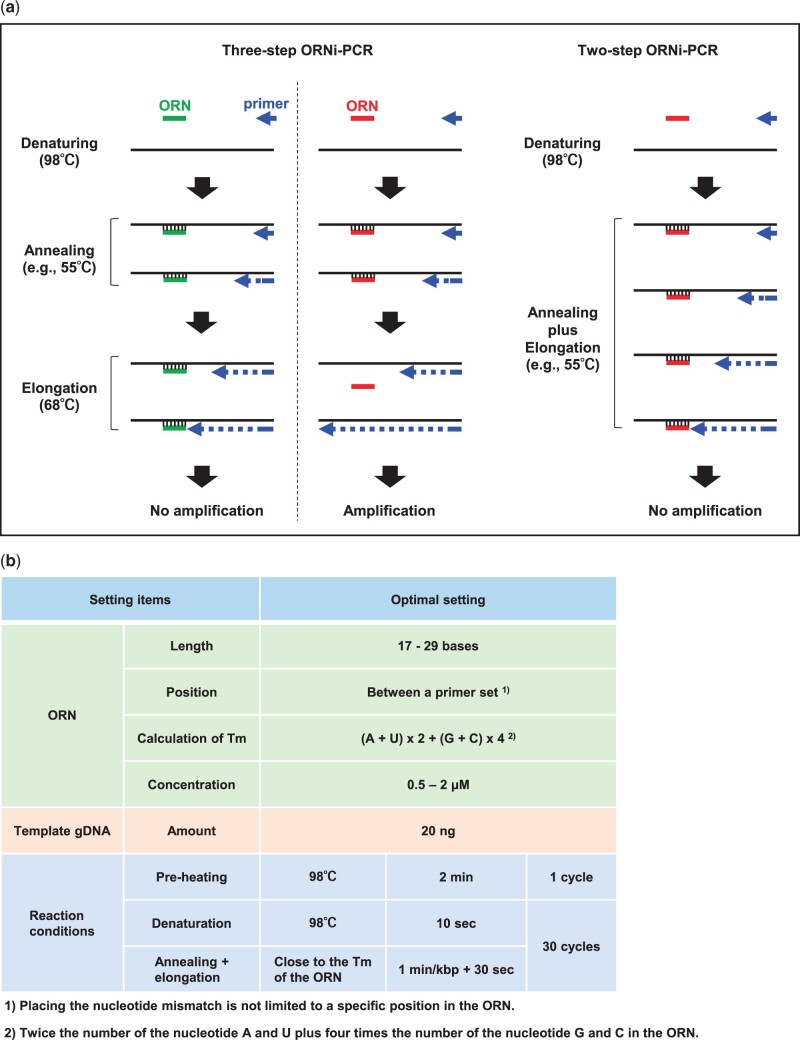
Schematic diagram of two-step ORNi-PCR. (a) Comparison of three-step and two-step ORNi-PCR. In three-step ORNi-PCR, if the Tm of an ORN (left panel) is higher than the elongation step temperature, the ORN can hybridize to the target DNA sequence, even during the elongation step, resulting in no amplification. If the Tm of an ORN (right panels) is lower than the elongation step temperature, the hybridized ORN detaches from the target DNA during the elongation step, resulting in amplification of the target region. In two-step ORNi-PCR, the temperature for the annealing and elongation steps is the same, and both steps are performed at a temperature lower than the Tm of the ORN. Thus, the ORN hybridizes stably to the target DNA sequence during the annealing and elongation steps, resulting in no amplification. (b) Technical information about two-step ORNi-PCR. Modified from Figures 3 and 4 in Fujita *et al*. [10]. Reused here under the Creative Commons Attribution (CC BY) License.

Subsequently, we established a two-step ORNi-PCR protocol (Fig. 2) [10]. Differences in the suppression modes of three-step and two-step ORNi-PCR are described in the legend to Fig. 2a. Selection of three-step or two-step ORNi-PCR is dependent on the sequence (i.e., Tm value) of an ORN. In this regard, and following the general rule about design of an ORN [10], the Tm value of an ORN will be around 60–68°C, which is not higher than the general elongation temperature of three-step PCR (around 68°C) for α-type DNA polymerases. Therefore, considering undesirable detachment of a hybridized ORN during the elongation step of three-step PCR, two-step ORNi-PCR will be the first choice. We have already established an optimization protocol for two-step ORNi-PCR (Fig. 2b). Following the protocol, one can perform two-step ORNi-PCR smoothly. We showed the practical utility of two-step ORNi-PCR. For example, the two-step ORNi-PCR can discriminate single-nucleotide mutations in gDNA generated by genome editing, or mutations in cDNAs from cancer cells [10]. We also showed that two-step ORNi-PCR can detect a single-nucleotide mutation in a target locus in cancer cells, even in the presence of a large number of cells with WT loci, and even if cancer cells account for only 0.2% of the total cell population [10].

## Using ORNi-PCR to amplify target splice variants

**Figure 3:**
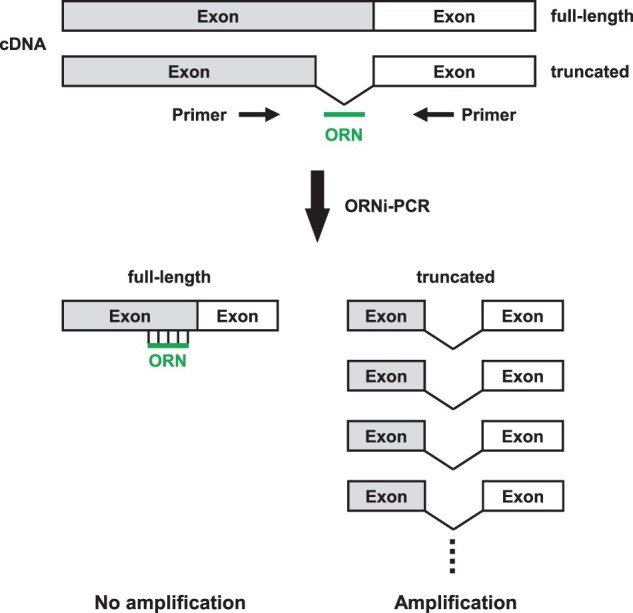
Schematic diagram showing discrimination of splice variants by ORNi-PCR of cDNA. Two forms of transcript (full length and truncated) are produced. An ORN is designed to be complementary to the full-length form. Amplification of the full-length transcript is completely suppressed by the ORN, but that of the truncated form is not.

Previously, we examined whether ORNi-PCR is capable of sequence-specific suppression of target amplification, even when cDNA is used as a template. We showed that the transcript of chicken *Pax5-1B* in DT40 (a chicken B cell line) has two splice variants (full length and truncated) [11]. We used an ORN complementary to the full-length form (the target region does not exist in the truncated form) for ORNi-PCR (Fig. 3). Amplification of full-length *Pax5-1B* was suppressed completely by the ORN; however, that of the truncated form was not [10]. These results suggest that ORNi-PCR can amplify a desired transcript variant from cDNA by suppressing amplification of undesirable transcripts.

## Use of ORNi-PCR to detect two mutations simultaneously

**Figure 4:**
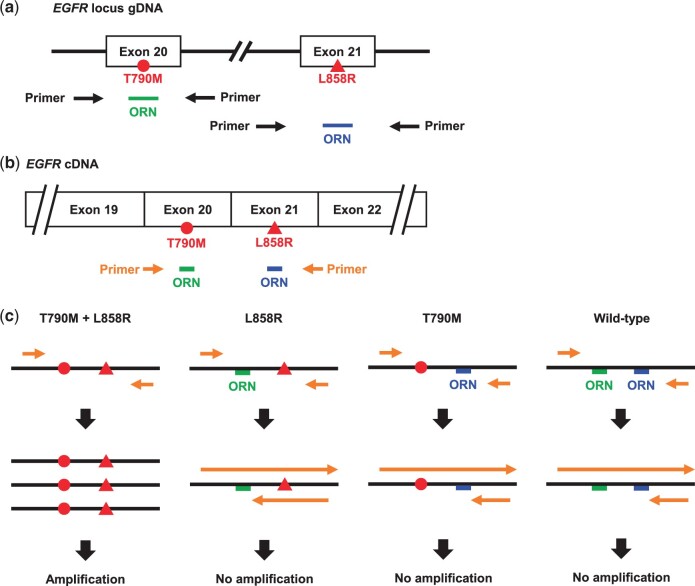
Schematic diagram showing simultaneous detection of the T790M and L858R mutations in gDNA and cDNA by ORNi-PCR. (a) The clinically significant single-nucleotide mutations in the human *EGFR* gene. T790M and L858R are located in exons 20 and 21, respectively. An ORN designed to detect the T790M mutation is shown in green, and an ORN targeting the L858R mutation is shown in blue. The primer positions are shown by arrows. The T790M and L858R mutations are represented by the circle and triangle, respectively. (b) Primer positions for ORNi-PCR of cDNA. The ORNs were used simultaneously. (c) Schematic diagram of ORNi-PCR using cDNA. If both mutations are present in the EGFR cDNA, the ORNs cannot hybridize to the cDNA and the target is amplified. If either of the two mutations (not both) are present in the cDNA, or neither of them is present, an ORN hybridizes with the cDNA and suppresses amplification of the target. The T790M and L858R mutations are represented by the circle and triangle, respectively. Modified from Figures 3d, 4a, and 4b in Baba *et al*. [19]. Reused here under the Creative Commons Attribution (CC BY) License.

Tyrosine kinase inhibitors (TKIs) are used as molecular targeted therapy for intractable diseases such as cancer [12]. Identification of *EGFR* gene mutations in lung cancer cells is important because certain types of *EGFR* gene mutations confer resistance to EGFR-TKIs, and the choice of EGFR-TKIs treatment is determined by the type of *EGFR* gene mutation. In lung cancer, for example, some amino acid mutations in EGFR encoded by the driver *EGFR* gene have been identified, including Leu858Arg (L858R), Thr790Met (T790M), and a partial amino acid deletion encoded by exon 19 (Ex19Del) [13]. First-generation (Erlotinib and Gefitinib) and second-generation (Dacomitinib and Afatinib) EGFR-TKIs are used to treat cancers harboring L858R and Ex19Del; T790M is observed usually in cancers that have acquired drug resistance due to selective pressure during clinical care [14, 15]. The presence of T790M is the norm with respect to prescription of Osimertinib, a third-generation EGFR-TKI [16–18]. Therefore, to detect such *EGFR* gene mutations, simple and reliable methods are required. Thus, we attempted rapid and simultaneous detection of these mutations by ORNi-PCR. To do this, we used gDNA from 293 T cells that harbor the WT *EGFR* gene, and gDNA from NCI-H1975 [a human lung cancer cell line harboring the T790M (C2369T) and L858R (T2573G) single-nucleotide mutations in the same allele]. We designed two ORNs that are complementary to the WT sequence of the region that harbors either the T790M or the L858R mutation, and designed primers to amplify target sequences across the T790M and L858R mutations (Fig. 4a). Amplification of both regions was completely suppressed by ORNs when 293 T gDNA was used, but not when NCI-H1975 gDNA was used [19].

Next, we examined whether ORNi-PCR enables simultaneous detection of the L858R and T790M mutations in cDNA. For this experiment, the ORN does not hybridize to the mutated template, and the target region is amplified only when both the T790 and L858 positions are mutated (i.e., T790M and L858R) (Fig. 4b and c). PCR amplified the target region within the WT *EGFR* gene; however, ORNi-PCR using ORNs specific for T790M or L858R amplified the target region in NCI-H1975 cDNA only [19].

We also examined the mutant-to-WT ratio (sensitivity) of ORNi-PCR for gDNA and cDNA. First, 293 T gDNA was mixed with NCI-H1975 gDNA and used as an ORNi-PCR template. Multiplex ORNi-PCR using ORNs specific for T790M and L858R suppressed amplification of the WT *EGFR* sequence from 293 T gDNA. In contrast, when NCI-H1975 gDNA accounted for 1% of total gDNA, the mutated *EGFR* sequences were amplified in the presence of the ORNs, but the WT *EGFR* sequences were not. We also mixed cDNA harboring WT *EGFR* sequences with NCI-H1975 cDNA and used it as a template. ORNi-PCR suppressed amplification of the WT *EGFR* sequence. In contrast, the mutated *EGFR* sequences were amplified in the presence of the ORNs, when NCI-H1975 accounted for >0.2% of the total cell number [19]. Taken together, these results suggest that ORNi-PCR can detect two single-nucleotide mutations simultaneously in the same allele of the *EGFR* gene with high sensitivity.

## ORNi-PCR using formalin-fixed paraffin-embedded specimen DNA

The organic solvent formalin is used widely for strong fixation of clinical tissue specimens. However, in general, DNA prepared from fixed tissue specimens is not suitable for PCR analysis because fixation by organic solvents causes DNA fragmentation [20–23]. Since histopathological formalin-fixed paraffin-embedded (FFPE) specimens are often used for diagnosis, it is important to see whether single-nucleotide mutations can be detected by ORNi-PCR using DNA templates obtained from FFPE specimens. We used FFPE specimens from a human cell line harboring the WT *EGFR* gene, and from cell lines harboring the WT *EGFR* gene as well as single-nucleotide mutations of the *EGFR* gene, including the L858R mutation. DNAs extracted from these specimens were used as a template for ORNi-PCR. The *EGFR* gene harboring L858R was detected by ORNi-PCR, even though it constituted only 5% of all *EGFR* genes [24]. However, the sensitivity of ORNi-PCR for DNA extracted from FFPE specimens was ∼30-fold lower than for gDNA extracted from cultured human cells [10], probably because the DNA was severely damaged by strong formalin fixation [20–23]. The use of a PNA-mediated PCR clamping method and clinical specimens (frozen sections) from lung cancer patients revealed that the sensitivity for detecting the *KRAS* gene mutation is 1% [25]. Another report showed that when a PNA-mediated PCR clamping method was used with plasma cell-free DNA, the sensitivity for detecting an *EGFR* gene mutation is 0.1% [26]. Therefore, further improvements are needed to increase the sensitivity of ORNi-PCR for DNA sequences in clinical samples.

## ORNi-PCR using whole blood specimens without prior DNA purification

Whole blood specimens are used widely for clinical diagnosis. In general, DNA is extracted from whole blood samples prior to analysis by PCR; the extraction procedure removes known and unknown substances that potentially inhibit PCR reactions. Alternatively, specialized PCR reagents enable PCR using whole blood samples. In this context, we used purified DNA as a template for ORNi-PCR. However, ORNi-PCR would be a more convenient clinical diagnostic method if whole blood specimens could be used directly (i.e., without DNA extraction) as ORNi-PCR templates. Therefore, we examined the use of whole blood from a rat as an ORNi-PCR template without prior DNA extraction. For this, whole blood from a Sprague–Dawley rat (GA (^198^Lys (AAG)-^199^Ser (AGC))-type *Gstm1* encoding glutathione S-transferase mu 1) and a Hirosaki hairless rat (TT (^198^Asn (AAT)-^199^Cys (TGC))-type *Gstm1*) was collected and used as an ORNi-PCR template to detect the *Gstm1* polymorphism [27]. We used KOD FX (Toyobo) as the DNA polymerase for ORNi-PCR because this reagent can efficiently amplify target DNA in crude samples such as tissue lysates or blood [28]. In the presence of an ORN corresponding to the GA-type sequence, amplification of GA-type *Gstm1* was suppressed, whereas TT-type *Gstm1* was amplified [24]. These results suggest that whole blood specimens can be used directly as ORNi-PCR templates to discriminate nucleotide differences without prior DNA extraction. It would be an interesting future issue to examine whether ORNi-PCR is compatible with commercial PCR kits designed to use whole blood specimens as templates, which may further expand the utility of ORNi-PCR.

## Detection of DNA methylation status by ORNi-PCR

**Figure 5:**
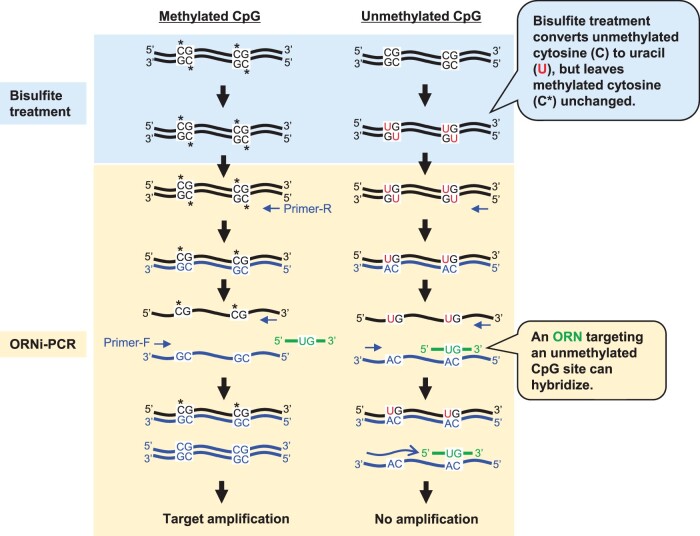
Schematic diagram showing detection of DNA methylation status by ORNi-PCR. ORNi-PCR using an ORN hybridizing with the converted target sequence (unmethylated cytosine to uracil) is performed using bisulfite-treated DNA as a template. An ORN that targets unmethylated CpG sites hybridizes with a corresponding DNA sequence synthesized during the first cycle of ORNi-PCR, thereby inhibiting amplification of the target. Modified from Figure 5 in Shimizu *et al*. [24]. Reused here under the Creative Commons Attribution (CC BY) License.

Detection of DNA methylation is important for clinical diagnosis because not only DNA sequence mutation, but also DNA methylation, plays a role in carcinogenesis [29]. To detect CpG methylation status, bisulfite-treated DNA is used widely as a PCR template. However, bisulfite treatment (which converts cytosine to uracil) damages DNA, making it difficult to use as a PCR template [30, 31]. Therefore, we investigated whether bisulfite-treated DNA could be used for ORNi-PCR. Unmethylated cytosines are converted to uracil by bisulfite treatment, whereas methylated cytosines remain unchanged (Fig. 5). We designed two types of ORN to target methylated and unmethylated CpG sites in the human *CDKN2A (p16)* gene. We extracted gDNA from a human colon cancer cell line, HCT116, treated it with bisulfite, and then used it as an ORNi-PCR template. The *CDKN2A (p16)* genes in HCT116 are CpG-methylated on one allele but not the other [32]. Amplification of the target *CDKN2A (p16)* sequence in the bisulfite-treated methylated and unmethylated alleles of HCT116 was suppressed strongly by ORNi-PCR using ORNs targeting methylated and unmethylated CpG sites, respectively [24]. These results suggest that the CpG methylation status of a target sequence within bisulfite-treated DNA can be detected by ORNi-PCR.

## Use of ORNi-PCR to enrich target DNA from a DNA mixture

**Figure 6:**
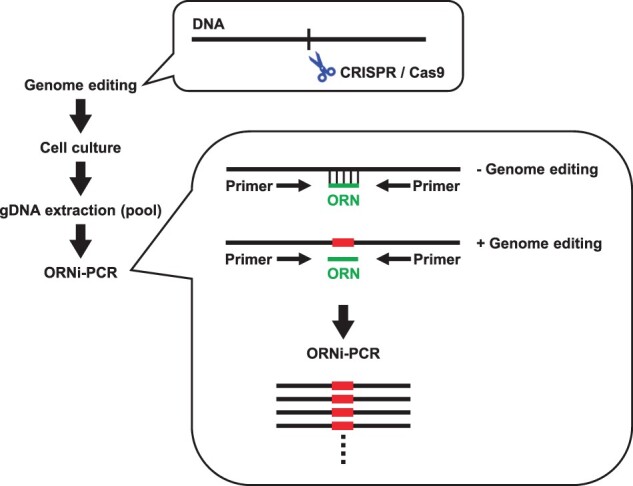
Schematic diagram showing enrichment of genome-edited DNA sequences by ORNi-PCR. Genome editing was performed using the CRISPR/Cas9 system, and gDNA prepared from the pool of genome-edited cells was used as a template for ORNi-PCR. Amplification of nonedited gDNA (− Genome editing) is suppressed by an ORN hybridizing to the cleavage site of the CRISPR/Cas9 system. In contrast, genome-edited gDNA (+ Genome editing) is enriched and amplified.

When analyzing the types of insertion/deletion (indel) mutations introduced by genome editing, PCR is used to amplify DNA sequence across a target site. The PCR products are cloned and then sequenced using the Sanger method. In this context, if amplification of nonedited (i.e., WT) DNA sequences is suppressed, it would reduce the effort and cost required for such analyses. Suppressing amplification of nonedited DNA sequences is important for detecting mutations, especially when the percentage of genome-edited cells is low. Therefore, we tried to enrich genome-edited DNA sequences by suppressing nonedited DNA sequences using ORNi-PCR. We used the clustered regularly interspaced short palindromic repeats (CRISPR) system/Cas9 as a genome editing tool to introduce mutations into the human *thymocyte nuclear protein 1* (*THYN1*) locus of HCT116 cells [33, 34]. After genome editing, we extracted gDNA from the pool of genome-edited cells and used them for ORNi-PCR (Fig. 6). For this, we designed an ORN complementary to the sequences around the cleavage site of the CRISPR system. Amplification of the *THYN1* locus was suppressed completely by the ORN when gDNA from WT HCT116 cells was utilized as an ORNi-PCR template. In contrast, amplification of the *THYN1* locus was detected even in the presence of the ORN when gDNA from genome-edited HCT116 cells was utilized as a template. DNA sequencing analysis showed that only WT *THYN1* sequences were detected in the PCR products when the ORN was absent from the PCR reaction solution, whereas genome-edited DNA sequences were detected from ORNi-PCR products. Cloning and sequencing of these amplicons revealed that edited *THYN1* sequences were detected in all 7 ORNi-PCR clones, but in only two of 11 PCR clones in the absence of ORNi-PCR [35]. These results suggest ORNi-PCR is useful for confirming the presence or absence of edited DNA sequences (i.e., the success or failure of genome editing), and enrichment of genome-edited sequences, by suppressing amplification of nonedited sequences.

**Figure 7:**
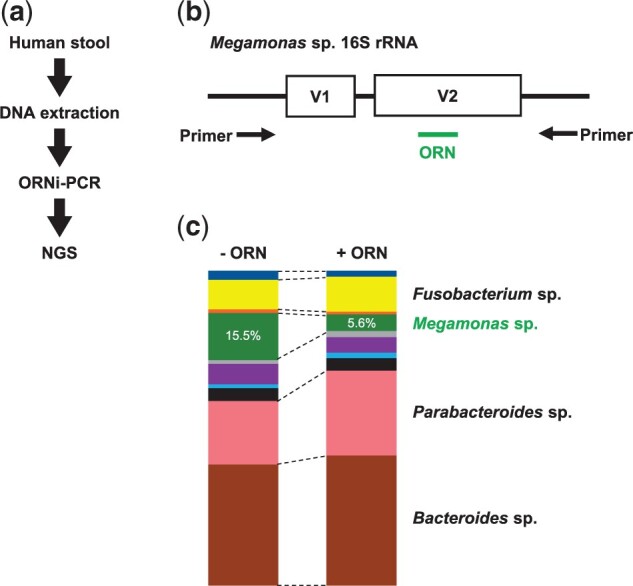
Suppression of target bacterial *16S rRNA* gene amplification using ORNi-PCR. (a) Flow of *16S rRNA* gene analysis by ORNi-PCR. (b) The ORN targeting the *Megamonas* sp. *16S rRNA* gene. The V1 and V2 region of *16S rRNA* gene were amplified using a common bacteria primer set. (c) Results of NGS analysis. After purification of PCR and ORNi-PCR amplicons, they were used for NGS analysis. Modified from Figure 3a, b, and e in Fujita *et al*. [35]. Reused here under the Creative Commons Attribution (CC BY) License.

PCR is used to amplify variable regions within bacterial *16S ribosomal RNA* (*16S rRNA*) genes for microbiome profiling, and the PCR products are analyzed by NGS. *16S rRNA* gene-based microbiome profiling of the human gut detected an abundance of genes from several dominant bacterial genera [36]. *16S rRNA* genes from less dominant genera might be amplified more abundantly if those from dominant genera can be suppressed by ORNi-PCR, allowing more detailed analysis without increasing the NGS read number. Therefore, we investigated whether amplification of a *16S rRNA* gene from a target bacterial genus in a human gut microbiome could be suppressed by ORNi-PCR. We prepared a primer set to amplify the V1–V2 regions of bacterial *16S rRNA* genes, and an ORN targeting the V2 region DNA sequences of the *16S rRNA* gene from *Megamonas* sp. (Fig. 7a and b). We carried out ORNi-PCR using DNA prepared from human stools as a template, and used the ORNi-PCR products for NGS analysis. We found that the amount of the *Megamonas* sp. *16S rRNA* gene fell from 15.5% to 5.6%. This result demonstrates that ORNi-PCR suppresses amplification of a *16S rRNA* gene during *16S rRNA* gene-based microbiome profiling. In addition, the amount of *16S rRNA* from some minor and other major bacterial genera increased (Fig. 7c) [35], suggesting that ORNi-PCR is useful for detecting genes from less dominant genera by suppressing genes from dominant genera without increasing the NGS read number. Suppression of the *16S rRNA* gene from *Megamonas* sp. by ORN was incomplete in this experiment; therefore, further analyses (such as optimization of experimental conditions) are required.

## Conclusions

ORNi-PCR has the potential for use not only in molecular biology, but also in various fields such as healthcare and agriculture. ORNs are economical to synthesize; therefore, they are more cost-effective than artificial nucleic acids such as PNAs and LNAs, and can be used for various applications. In addition, ORNs can detect nucleotide differences when used for isothermal DNA amplification reactions such as recombinase polymerase amplification [37]. Technology based on ORNs, including ORNi-PCR, is expected to be useful for early detection of cancers caused by single-nucleotide mutations because it will speed up the process and increase sensitivity.

## Author Contributions

All authors participated in the writing and editing of the article.

## Funding

This research received no external funding.

## Conflict of interest statement 

T.F. and H.F. hold the following patents for ORNi-PCR: (i) name: Method for suppressing amplification of specific nucleic acid sequences; patent numbers: Japan 6,928,323 and 6,964,900; (ii) name: Method for detecting differences in target nucleic acid region; patent publication number: WO 2019/203350; patent numbers: Japan 6,653,932, Canada 3,096,462, Korea 10-2,285,085, Israel 277,764, and the US 11,155,873. Epigeneron, Inc. owns the rights to commercial use of ORNi-PCR. T.F. and H.F. are founders of Epigeneron, Inc. and own stock in the company. H.F. is one of directors of Epigeneron, Inc., and T.F. is one of its advisers.

## Data availability statement

There is no new data associated with this review manuscript.
